# Pre-procedural disclosure of potential diagnostic errors in radiology: public perspectives in the Netherlands

**DOI:** 10.1007/s00330-026-12656-8

**Published:** 2026-06-03

**Authors:** Thomas C. Kwee, Yfke Ongena, Marieke Haan, Derya Yakar

**Affiliations:** 1 Department of Radiology, Medical Imaging Center, University Medical Center Groningen, University of Groningen, Groningen, The Netherlands; 2 Center for Language and Cognition, Discourse and Communication Group, University of Groningen, Groningen, The Netherlands; 3 Department of Sociology/ICS, Faculty of Behavioral and Social Sciences, University of Groningen, Groningen, The Netherlands

**Keywords:** Diagnostic errors, Disclosure, Patient preference, Radiology, Risk assessment

## Abstract

**Objective:**

To assess public attitudes toward prospective disclosure of diagnostic error risk in radiologic imaging (i.e., informing patients in advance about the possibility a radiologic examination may yield an incorrect finding, rather than disclosure of an error after it has occurred) and to identify factors influencing these preferences.

**Materials and methods:**

A population-based survey was conducted in the Netherlands. Participants aged ≥ 18 years (*n* = 1524) responded to CT-scan vignettes describing pre-procedural potential diagnostic errors, varying by patient age (9, 26, 46, or 71 years) and probability of incorrect findings (< 0.1%, < 1%, < 5%, or unspecified). For case vignettes involving 9-year-old patients, participants responded as parents/guardians. Agreement with “I would want to be informed” was measured using a 4-point Likert scale (1 = strongly disagree, 2 = disagree, 3 = agree, 4 = strongly agree).

**Results:**

Overall, 88.4% of participants agreed or strongly agreed that patients should be informed about the risk of diagnostic errors. Patient age and diagnostic error risk significantly influenced agreement. Compared with vignettes describing a 71-year-old patient, younger patient ages were associated with higher odds of agreement (9-year-old OR 4.023, *p* = 0.010; 26-year-old OR 3.222, *p* = 0.019; 46-year-old OR 2.659, *p* = 0.022). Compared with vignettes in which diagnostic error risk was unspecified, specifying risks of < 0.1% or < 1% was associated with lower odds of agreement (< 0.1% risk: OR 0.476, *p* < 0.001; < 1% risk: OR 0.635, *p* = 0.002), whereas a < 5% risk was not (OR 0.876, *p* = 0.360).

**Conclusion:**

Public support for prospective disclosure of diagnostic error risk in radiologic imaging is high in the Dutch population, particularly for younger patients and higher-risk scenarios.

**Key Points:**

***Question*** *Most adults in the Netherlands support disclosure of diagnostic error risk in radiologic imaging, particularly for younger patients and higher-risk scenarios*.

***Findings*** *Public preferences for disclosure are driven by patient age and error probability, rather than participant demographics or prior radiology experience*.

***Clinical relevance*** *Public support for disclosure of diagnostic error risk in radiologic imaging is high in the Dutch population, especially for younger patients and higher-risk scenarios*.

**Graphical Abstract:**

**Figure:**
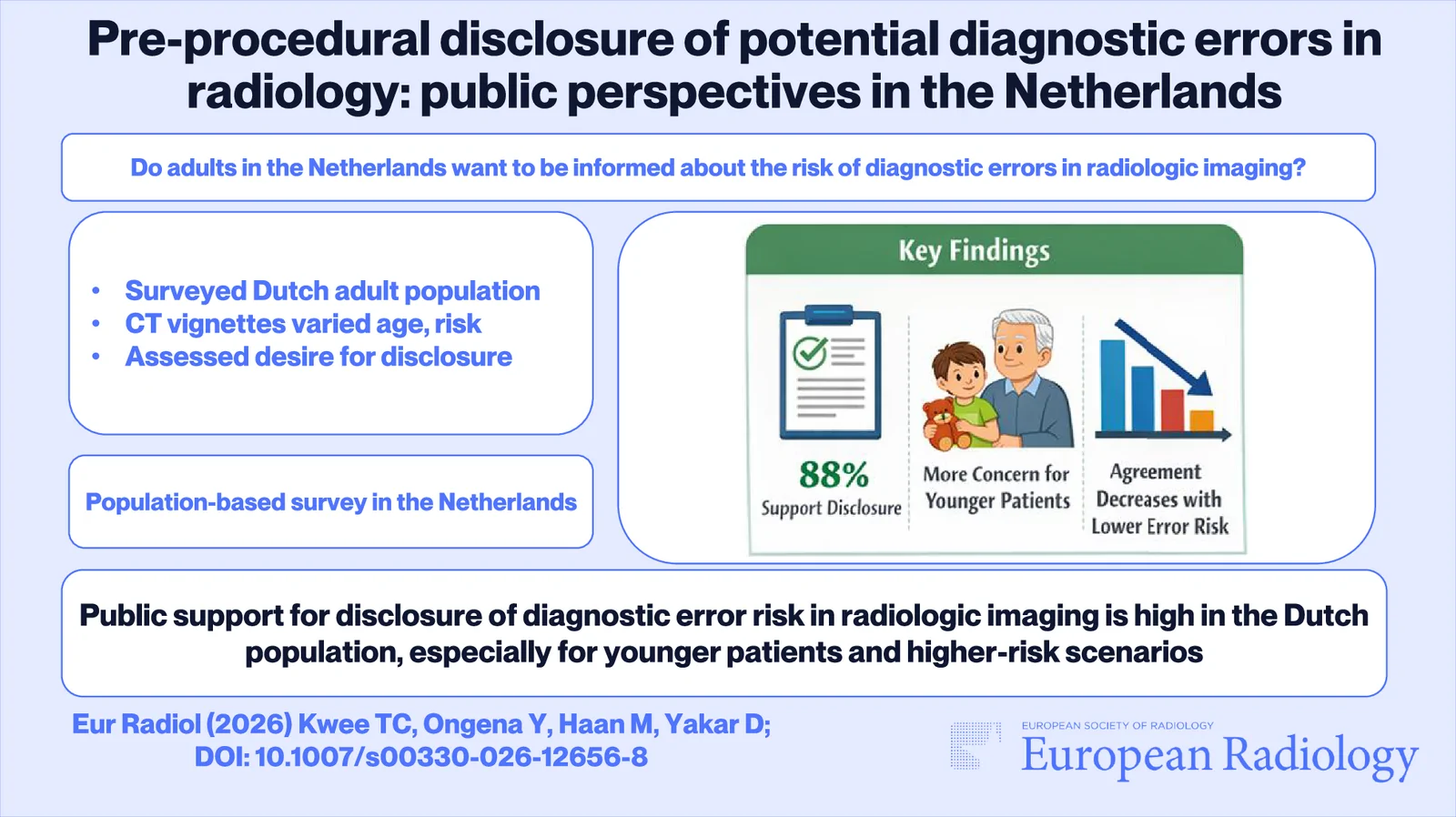

## Introduction

Diagnostic imaging plays a central role in modern medicine, guiding clinical decision-making across a wide range of conditions. Despite substantial advances in imaging technology, workflow optimization, and interpretive support systems, diagnostic errors in radiology remain an important patient-safety concern. These errors, commonly categorized as perceptual misses or interpretive inaccuracies, are estimated to occur in approximately 3–5% of routine imaging studies (approximately 30% if only the number of abnormal imaging results is used as the denominator), potentially leading to delayed or missed diagnoses, misdiagnoses, inappropriate or harmful treatment, and increased healthcare utilization [1–4], with patients often discovering these errors only after persistent symptoms or adverse outcomes.

An important but underexplored aspect of diagnostic error in radiology is the perspective of patients. As healthcare systems increasingly emphasize transparency, shared decision-making, and patient empowerment, questions arise regarding how much information patients want about the possibility of diagnostic error in their imaging studies. While clinicians and policymakers debate the benefits and risks of disclosure (balancing ethical obligations, medicolegal implications, and potential patient anxiety), the preferences of the general population remain insufficiently understood [4]. In this study, we focus on prospective disclosure of diagnostic error risk, defined as informing patients before imaging about the possibility that an incorrect finding may occur. This concept differs from retrospective error disclosure, which involves informing patients after a diagnostic error has already taken place. While retrospective error disclosure has been more widely studied, prospective risk disclosure remains underexplored.

To address this gap, we conducted a population-based survey to assess public attitudes toward prospective disclosure of diagnostic error risk in radiologic imaging. Our aim was to characterize the level of interest in such information, identify factors associated with preferences for disclosure, and provide evidence to inform patient-centered communication strategies in radiology.

## Materials and methods

### Study design and participants

Data for this study were drawn from the September 2022 round of the Longitudinal Internet Studies for the Social Sciences (LISS) Panel, an online survey panel designed to provide a nationally representative sample of the Dutch population aged sixteen and older. For the present analysis, we restricted the sample to adults aged eighteen and above to focus on individuals capable of independently forming and expressing their views on prospective disclosure of diagnostic error risk, avoiding potential influence from parental guidance or limited healthcare experience in younger respondents. Furthermore, only one respondent per household was included when multiple household members were enrolled. Participation in the LISS Panel follows a two-step informed consent procedure involving both a returned reply card and verification through an online login [5]. The LISS Board of Overseers reviewed and approved the panel’s data collection procedures. Surveys could be completed on a desktop computer, tablet, or smartphone.

### Survey

Participants were provided with a brief introduction to radiology and its potential advantages and disadvantages (see Supplementary file 1 for the full text). The questionnaire was administered entirely in Dutch and was available online through the LISS Panel website. As part of standard LISS Panel procedures, the questionnaire (including the vignettes) was reviewed by Centerdata for clarity and comprehensibility and required to meet an accessible language level (approximately B1) to ensure suitability for the general population [6]. To investigate the general population’s view on the need for disclosure regarding the risk of diagnostic errors in radiologic imaging, 13 case vignettes (hypothetical scenarios [7]) were included in the September 2022 data collection. Vignettes were developed collaboratively by two radiologists (T.C.K. and D.Y.) and two survey specialists (Y.O. and M.H.) through an iterative process. Draft vignettes were reviewed and refined multiple times to ensure clarity, consistency, and comprehensibility for a general population audience. All vignettes were designed to reflect prospective communication prior to imaging, focusing specifically on disclosure of the risk of diagnostic error rather than disclosure of an error that has already occurred. Each vignette described a patient who was ill and required a CT scan to visualize the disease as accurately as possible for better treatment. All vignettes were based on the same core scenario, with differences introduced only in patient age and the probability of an incorrect CT finding, while all other elements remained identical. Patient age was systematically varied (9, 26, 46, or 71 years) to represent distinct life stages, including childhood, young adulthood, middle age, and older adulthood, and to explore whether preferences for disclosure differed across the lifespan. The risk of an incorrect CT finding was set at < 0.1%, < 1%, < 5%, or unspecified. The < 5% cap was chosen because routine imaging audits generally show discrepancy rates below 5% [8]. For the 9-year-old patient case, respondents were instructed to answer from the perspective of a parent or legal guardian. Note that a total of 16 different vignettes could have been created from the combination of four patient age groups and four risk categories. However, only 13 vignettes were included in the survey, using a structured sampling strategy [9] to ensure all key aspects were represented while keeping the task manageable for respondents, as the survey also contained multiple questions unrelated to diagnostic error. This reduction was not based on a formal power calculation but was part of a broader survey design approach in which similar reductions were applied across multiple vignette-based sections to limit overall questionnaire length and complexity. The three excluded vignettes (9-year-old with < 1% risk, 26-year-old with < 5% risk, and 71-year-old with unspecified risk) were removed to reduce respondent burden while maintaining coverage of all key age and risk combinations. Participants were randomly assigned to one to four vignettes. In total, all participants received 10 vignettes, but the remaining vignettes covered other topics unrelated to disclosure of diagnostic errors. The vignettes were presented in a randomized order to eliminate potential order effects, ensuring that responses to a vignette about a young patient, for example, would not systematically influence responses to a vignette about an older patient later in the survey. An example vignette is as follows: “A patient is 46 years old and ill. The doctors want to visualize the disease as accurately as possible to provide better treatment. The CT scan that the patient needs for this can result in an incorrect finding in less than 5% of cases.” Following each vignette, participants were then asked: “Please review the situation above. Do you disagree or agree with the following statement: If I were in this situation, I would want to be informed.” Responses were recorded on a 4-point Likert scale (1 = strongly disagree, 2 = disagree, 3 = agree, 4 = strongly agree, or don’t know). The “don’t know” option is not a midpoint of the scale but an explicit choice for participants who felt unable to decide or had no opinion. Higher numbers consistently indicate greater agreement, and this coding was applied uniformly across all vignettes, as the statement wording was always the same.

### Statistical analysis

Descriptive statistics were first used to summarize participants’ responses, indicating the proportion of those who agreed or disagreed that prospective disclosure of diagnostic error risk in radiologic imaging should be provided to patients. Responses were reported as percentages for the overall sample. Descriptive analyses were then stratified by the patient’s age in the vignettes and by the level of risk of diagnostic error presented in each vignette.

To examine factors associated with agreement, we conducted a multivariate ordinal regression analysis using survey participants’ age, self-reported gender, educational level, prior experience with radiologic procedures, and the patient’s age and risk of diagnostic error as indicated in each vignette as predictor variables. Survey participants’ age, self-reported gender, and educational level were included because these factors may influence preferences for disclosure. Older adults may perceive medical risks differently and may have different expectations for information compared with younger adults. Gender differences may affect health-related preferences, communication styles, and risk perception, which could influence the desire to be informed about diagnostic errors. Educational level may influence survey participants’ understanding of medical information, which could affect disclosure preferences. Likert-scale responses were treated as ordered categories in the regression models. Because participants could respond to multiple vignettes, we assessed potential clustering of responses at the participant level by calculating the intraclass correlation coefficient (ICC) to inform the choice between a standard ordinal regression model and a mixed-effects ordinal regression model with participant as a random effect. This ICC assessment was performed exclusively for the vignette questions on pre-procedural disclosure of potential diagnostic errors in radiology. Variables with unknown or missing data, “other” response categories, categories with fewer than 10 observations, and “don’t know” responses were excluded from the regression analysis.

Statistical significance was set at a two-sided *p*-value of < 0.05. Statistical analysis was performed using IBM SPSS Statistics version 30.0 (IBM Corporation).

## Results

### Participants

In September 2022, 3,425 members of the LISS panel were invited to participate, and 2407 completed the questionnaire (response rate 70.3%). Of the 2407 participants who completed the questionnaire, 1524 were randomly assigned to one to four case vignettes, resulting in a total of 2435 vignette responses addressing disclosure of the risk of diagnostic errors in radiologic imaging (mean, 1.6 vignettes per participant). Note that all 2,407 participants received a total of 10 vignettes, but the remaining vignettes covered other topics, with between one and four vignettes per participant specifically focused on disclosure of diagnostic error risk. The ICC for responses across vignettes from the same participant was 0, indicating no between-participant variance and that a standard multivariate ordinal regression model was appropriate [10]. Participants’ median age was 51 years (range: 18–96 years); 53.0% were female, 50.4% had completed secondary education, and 73.9% had previously undergone a radiologic procedure (Table 1).

**Table 1:** Characteristics of included participants (*n* = 1524)

Variable	Category	Number (%)^*^
Age (median and range)	NA	51 years (18–96 years)
Self-reported gender	Male	714 (46.8%)
	Female	807 (53.0%)
	Other	3 (0.2%)
Level of education^a^	Primary education	37 (2.4%)
	Secondary education^b^	768 (50.4%)
	Tertiary education^c^	675 (44.3%)
	Other^d^	44 (2.9%)
Prior exposure to radiologic procedures	Yes	1126 (73.9%)
	No	331 (21.7%)
	Unclear	67 (4.4%)

*NA* not applicable

^*^ Unless indicated otherwise

^a^ Highest earned degree

^b^ Pre-university education or mediated vocational education

^c^ University of higher vocational education

^d^ No degree or degree not included among response options

### Overall and stratified agreement on diagnostic error risk disclosure

Overall, the majority of participants (88.4%) agreed or strongly agreed that the risk of diagnostic errors in radiologic imaging should be disclosed to patients, while 8.8% disagreed or strongly disagreed, and 2.8% responded “don’t know” (Table 2). When stratified by the patient’s age in the vignettes, agreement was highest for the 9-year-old patient (91.7% agree/strongly agree) and lowest for the 71-year-old patient (84.3% agree/strongly agree) (Table 2). Stratification by the level of risk of diagnostic error indicated that participants were most likely to agree that consent should be obtained when the risk was < 5% (95.1% agree/strongly agree), and least likely when the risk was < 0.1% (80.9% agree/strongly agree) (Table 2).

**Table 2:** Participant agreement on obtaining disclosure for radiologic diagnostic error risks, based on 2435 case vignettes

Case vignette	Strongly disagree	Disagree	Agree	Strongly agree	Don’t know
All case vignettes, regardless of age and diagnostic error risk	1.7% (*n* = 41)	7.1% (*n* = 173)	34.2% (*n* = 832)	54.2% (*n* = 1320)	2.8% (*n* = 69)
Case vignettes describing a 9-year-old patient	0.3% (*n* = 2)	4.8% (*n* = 28)	32.5% (*n* = 188)	59.2% (*n* = 342)	3.1% (*n* = 18)
Case vignettes describing a 26-year-old patient	1.9% (*n* = 11)	6.8% (*n* = 40)	34.4% (*n* = 201)	54.3% (*n* = 317)	2.6% (*n* = 15)
Case vignettes describing a 46-year-old patient	0.9% (*n* = 6)	7.9% (*n* = 55)	34.6% (*n* = 240)	54.1% (*n* = 375)	2.5% (*n* = 17)
Case vignettes describing a 71-year-old patient	3.8% (*n* = 22)	8.6% (*n* = 50)	35.0% (*n* = 203)	49.3% (*n* = 286)	3.3% (*n* = 19)
Case vignettes describing a diagnostic error risk of < 0.1%	3.6% (*n* = 26)	12.6% (*n* = 90)	36.2% (*n* = 259)	44.7% (*n* = 320)	2.9% (*n* = 21)
Case vignettes describing a diagnostic error risk of < 1%	2.2% (*n* = 12)	9.0% (*n* = 50)	36.7% (*n* = 203)	49.2% (*n* = 272)	2.9% (*n* = 16)
Case vignettes describing a diagnostic error risk of < 5%	0.2% (*n* = 1)	3.0% (*n* = 17)	34.1% (*n* = 196)	61.0% (*n* = 350)	1.7% (*n* = 10)
Case vignettes with no absolute risk indicated	0.3% (*n* = 2)	2.7% (*n* = 16)	29.4% (*n* = 174)	63.9% (*n* = 378)	3.7% (*n* = 22)

Note that the full survey on diagnostic radiology consisted of 63 case vignettes, of which 13 addressed disclosure for diagnostic errors in radiologic imaging and are included in this analysis; the remaining vignettes covered other topics. Each participant evaluated six randomly assigned vignettes on diagnostic radiology, resulting in unequal numbers of responses across vignette categories. The 13 diagnostic error vignettes varied by patient age (9, 26, 46, or 71 years) and presentation of diagnostic error risk (< 0.1%, < 1%, < 5%, or no absolute risk specified). Because vignettes were randomly distributed and each participant reviewed only a subset, the number of observations differs across patient-age and risk categories. Percentages are calculated within each vignette subgroup

### Factors associated with agreement on diagnostic error risk disclosure

In multivariate ordinal regression analysis (Table 3), agreement with disclosure of diagnostic error risk was associated primarily with characteristics of the case vignettes, while participants’ age, self-reported gender, level of education, and prior exposure to radiologic procedures were not significantly associated with agreement. Compared with vignettes describing a 71-year-old patient, those describing a 9-year-old (odds ratio [OR] 4.023, *p* = 0.010), 26-year-old (OR 3.222, *p* = 0.019), and 46-year-old patient (OR 2.659, *p* = 0.022) were associated with higher odds of agreement, indicating that participants were more likely to endorse disclosure for younger patients. When considering the level of diagnostic error risk, participants were less likely to want disclosure if the risk was very low. Specifically, compared with vignettes with no stated risk, a risk of < 0.1% (OR 0.476, *p* < 0.001) or < 1% (OR 0.635, *p* = 0.002) was associated with a lower likelihood of participants wanting to be informed about the risk, whereas a risk of < 5% was not significantly different from the unspecified-risk condition (OR 0.876, *p* = 0.360).

**Table 3:** Multivariate analysis of variables associated with higher odds of endorsing disclosure of the risk of diagnostic errors

Variable	Category	OR	95% CI for OR	*p*-value
Participants’ age^a^	NA	1.001	0.996–1.006	0.811
Participant’s self-reported gender^b^	Male	1.029	0.871–1.217	0.733
Participants’ level of education^c,d^	Primary education	0.619	0.309–1.240	0.176
	Secondary education^e^	0.862	0.636–1.168	0.337
Participant’s prior exposure to radiologic procedures^f^	Yes	0.457	0.191–1.089	0.077
Patient’s age^g,h^	9 years	4.023	1.397–11.600	***0.010***
	26 years	3.222	1.213–8.551	***0.019***
	46 years	2.659	1.153–6.141	***0.022***
Risk of diagnostic errors^i^	< 0.1%	0.476	0.354–0.640	***< 0.001***
	< 1%	0.635	0.478–0.845	***0.002***
	< 5%	0.876	0.660–1.163	0.360

Significant *p*-values are shown in bold italics

*CL* confidence interval, *NA* not applicable

^a^ Continuous variable

^b^ Female gender was used as the reference category

^c^ Highest earned degree

^d^ Tertiary education (university or higher vocational education) was used as the reference category

^e^ Pre-university education or mediated vocational education

^f^ No was used as the reference category

^g^ Patient’s age as indicated in each case vignette

^h^ 71 years was used as the reference category

^i^ No absolute risk was used as the reference category

## Discussion

In this nationally representative survey, the vast majority of participants (88.4%) supported informing patients about the risk of diagnostic errors prior to imaging. Importantly, this study addresses prospective risk disclosure rather than retrospective disclosure of actual diagnostic errors. Support for disclosure was higher for younger patients and in scenarios with a higher stated risk of diagnostic error, whereas survey participant demographics and prior experience with radiologic procedures were not associated with preferences for disclosure.

These findings have practical implications because diagnostic errors are relatively common in radiology, with retrospective studies suggesting rates up to 30% for abnormal imaging results and real-time errors averaging 3–5% [1, 2]. Some errors can lead to adverse outcomes, including malpractice claims [11, 12]. Strategies such as double reading, standardized reporting, and artificial intelligence support cannot fully eliminate errors, even among experienced radiologists [1, 13]. Although informed consent in the Netherlands focuses mainly on invasive or high-risk procedures, non-invasive imaging often relies on implied consent, and current procedures generally do not address diagnostic error risk, reflecting clinical conventions rather than absolute risk [4, 14]. Within the context of non-invasive imaging, our results show that survey participants value prospective disclosure of diagnostic error risk, which may support patient-centered care. Routine disclosure must balance transparency with feasibility, comprehension, and the potential for unnecessary anxiety or repeat testing. Fully individualized consent for diagnostic errors is challenging due to the high volume of imaging studies and variability in error types and probabilities [15, 16]. Specific, study-level error probabilities are generally unavailable, and many errors may not meaningfully affect patient management. A practical solution may involve brief, standardized information in brochures or consent forms, providing basic knowledge about the possibility of diagnostic errors without disrupting clinical workflow. We acknowledge that many patients may not read these materials, so this approach is intended to supplement, rather than replace, individualized communication and discussion when feasible. Future research should explore patient preferences for the format, depth, and timing of such information, and its impact on understanding, satisfaction, and decision-making. Similar errors can occur across other diagnostic tests and specialties, highlighting the broader relevance of patient-centered disclosure in medicine.

To our knowledge, there is little prior empirical research specifically addressing patient or public preferences for patient disclosure regarding the risk of diagnostic errors in radiologic imaging. While prior research has shown that disclosure of actual medical errors is generally associated with increased patient satisfaction and trust [17, 18], these findings may not directly apply to prospective disclosure of diagnostic error risk. In some cases, providing information about potential errors could increase anxiety or even reduce satisfaction, and the impact of risk disclosure on patient experience would need to be specifically studied in future research. A recent conceptual article highlighted that, although the principles of autonomy and informed consent support informing patients about the possibility of diagnostic errors, current practice generally does not include such disclosure for non-invasive imaging, and empirical data on public preferences are still lacking [4]. Our study provides novel quantitative data in this area by surveying a representative sample of the general population about their preferences for prospective disclosure of diagnostic error risk and identifying factors associated with these preferences. These findings, therefore, fill an important gap by moving from conceptual discussion to empirically grounded evidence about public attitudes toward diagnostic error disclosure in radiology, and by quantifying how preferences vary by patient age and stated risk level.

The present study had some limitations. First, the use of hypothetical case vignettes may not fully reflect patients’ preferences in real clinical situations, where emotional stress, illness severity, and time constraints could influence attitudes toward disclosure. Second, the vignettes provided limited clinical detail and presented diagnostic error as a general risk, without distinguishing between different types of errors, their clinical relevance, or potential consequences. Providing detailed, varied scenarios for each possible error would have been very difficult in a population-based survey and could have affected comprehension and response rates. Third, respondents’ understanding of the presented risk percentages (e.g., 5% = 1 in 20) may have varied, with comprehension generally higher among those with more education. However, as LISS panel members regularly complete surveys, they are likely more familiar with interpreting numerical information than the general population, which may slightly reduce representativeness for those who rarely complete surveys. It is possible that variation in understanding of risk magnitudes influenced participants’ responses, particularly in scenarios with very low probabilities, and this should be considered when interpreting the results. Fourth, although the sample was nationally representative of the Dutch population, findings may not be generalizable to other countries with different healthcare systems, legal frameworks, or cultural norms regarding disclosure practices.

In conclusion, public support for prospective disclosure of diagnostic error risk in radiologic imaging is high in the Dutch population, particularly for younger patients and higher-risk scenarios.

## Supplementary information

Supplementary information

## Abbreviations

LISS: Longitudinal internet studies for the social sciences
OR: Odds ratio
