# Supplementary material for: Pre-procedural disclosure of potential diagnostic errors in radiology: public perspectives in the Netherlands
Source: Eur Radiol. 2026 Jun 3;36(10):7632–8. doi: 10.1007/s00330-026-12656-8 (PMC13574909; doi:10.1007/s00330-026-12656-8)
Supplement: Supplementary file 1 [file 330_2026_12656_MOESM1_ESM.pdf]

ELECTRONIC SUPPLEMENTARY MATERIAL

Pre-procedural disclosure of potential diagnostic errors in radiology: public perspectives in the Netherlands

This questionnaire is about informed consent in radiology.
Radiology is a medical specialty that uses imaging examinations such as X-rays, ultrasounds, CT scans, and MRI scans. These examinations can be used to diagnose diseases and provide treatments (for example, opening blood vessels or removing tumors).

Possible benefits of radiology include:
- Accurate imaging allows for better prediction of disease progression and ensures appropriate treatment.
- Radiological treatments can reduce disease and mortality.
-

Possible risks of radiology include:
- X-rays and contrast agents can cause side effects (risk of cancer, allergic reactions, and organ damage).
- A disease may be incorrectly diagnosed.
- Abnormalities may be found that would never have caused any symptoms but could still be treated unnecessarily.
- Invasive procedures can lead to various complications (e.g., bleeding, collapsed lung, etc.).

Patients are legally required to give consent before undergoing a radiological imaging examination or treatment. They must assess for themselves whether the risks of an examination or treatment outweigh the benefits.
